# 

**Published:** 1866-12

**Authors:** 


### CONTENTS FOR DECEMBER.

ORIGINAL CONTRIBUTIONS.

Localized Bodily Movements: Their value as an aid in the treatment of Tuberculosis. By Robt. N. Tooker, M. D.......... 539

Clinical Lectures on Diseases of the Eye—Paralysis of the Muscles of the Eye. By E. L. Holmes, M. D.......... 550

A Case of Cerebro-Spinal Anæmia. By R. C. Hamill, M. D.......... 555

Poisoning with Morphine. Reported by I. L. Prentiss, M. D.......... 559

Observations on the "Clamp" in the treatment of Internal Hemorrhoids and Prolapsus of the Rectum, with Two Cases. By A. J. Baxter, M.D. 560

Enlargement of Lymphatic Glands about the Bifurcation of the Trachea and Bronchial Tubes, Obstructing Respiration. By C. Cushing, M.D. 564

PROCEEDINGS OF MEDICAL SOCIETIES.

Chicago Medical Society.......... 566

Morgan County Medical Society.......... 570

SELECTED ARTICLES.

Vivisection.......... 573

Successful Removal of a large Bronchocele. By Wm. Warren Greene, M. D.......... 576

EDITORIAL.......... 579

*Book Notices*—Practical Therapeutics, considered chiefly with reference to articles of the Materia Medica—A Handy Book of Ophthalmic Surgery for the use of Practitioners—Glaucoma and its cure by Iridectomy—Orthopœdics: A Systematic Treatise upon the Prevention and Correction of Deformities—A Practical Treatise on Diseases of the Skin—Cerebro-Spinal Meningitis: Being a Report made to the Illinois State Medical Society—An Introduction to Practical Chemistry—The Martyrs and Heroes of Illinois in the Great Rebellion.

Clinical Instruction in Chicago.......... 583

Rush Medical College.......... 584

City Mortality.......... 585

